# An optimal algorithm for computing all subtree repeats in trees

**DOI:** 10.1098/rsta.2013.0140

**Published:** 2014-05-28

**Authors:** T. Flouri, K. Kobert, S. P. Pissis, A. Stamatakis

**Affiliations:** 1 Heidelberg Institute for Theoretical Studies, 69118 Heidelberg, Germany; 2 King’s College London, London WC2R 2LS, UK; 3 Karlsruhe Institute of Technology, 76021 Karlsruhe, Germany

**Keywords:** tree data structures, unrooted unordered labelled trees, subtree repeats

## Abstract

Given a labelled tree *T*, our goal is to group repeating subtrees of *T* into equivalence classes with respect to their topologies and the node labels. We present an explicit, simple and time-optimal algorithm for solving this problem for unrooted unordered labelled trees and show that the running time of our method is linear with respect to the size of *T*. By unordered, we mean that the order of the adjacent nodes (children/neighbours) of any node of *T* is irrelevant. An unrooted tree *T* does not have a node that is designated as root and can also be referred to as an undirected tree. We show how the presented algorithm can easily be modified to operate on trees that do not satisfy some or any of the aforementioned assumptions on the tree structure; for instance, how it can be applied to rooted, ordered or unlabelled trees.

## 1. Introduction

Tree data structures are among the most common and well studied of all combinatorial structures. They are present in a wide range of applications, such as in the implementation of functional programming languages [1], term-rewriting systems [2], programming environments [3], code optimization in compiler design [4], code selection [5], theorem proving [6] and computational biology [7].

Thus, efficiently extracting the repeating patterns in a tree structure represents an important computational problem. Recently, Christou *et al.* [8] presented a linear-time algorithm for computing all subtree repeats in *rooted ordered unlabelled* trees. Christou *et al.* [9] extended this algorithm to compute all subtree repeats in *rooted ordered labelled* trees in linear time *and* space. The authors considered only *full subtrees*, i.e. subtrees which contain *all* nodes and edges that can be reached from their root.

The limitation of the aforementioned results is that they cannot be applied to *unrooted* or *unordered* trees. By unrooted, we mean that the input tree does not have a dedicated root node; and, by unordered, we mean that the order of the adjacent nodes (children/neighbours) of any node of the tree is irrelevant. Such trees are a generalization of rooted ordered trees, and, hence, they arise naturally in a broader range of real-world applications. For instance, unrooted unordered trees are used in the field of (molecular) phylogenetics [10,11].

*Biological motivation*. The field of molecular phylogenetics deals with inferring the evolutionary relationships among species using molecular sequencing technologies and statistical methods. Phylogenetic inference methods typically return unrooted unordered labelled trees that represent the evolutionary history of the organisms under study. These trees depict evolutionary relationships among the molecular sequences of extant organisms (living organisms) that are located at the tips (leaves) of those trees and hypothetical common ancestors at the inner nodes of the tree. With the advent of the so-called next-generation sequencing technologies, large-scale multi-national sequencing projects such as 1KITE^1^ (1000 insect transcriptome sequencing project) emerge. In these projects, large phylogenies that comprise thousands of species and massive amounts of whole-transcriptome or even whole-genome molecular data need to be reconstructed.

Provided there is a fixed multiple sequence alignment (MSA) of the sequences—representing species—under study, the goal of phylogenetic inference is to find *the* tree topology that best explains the underlying data, using a biologically reasonable optimality criterion—a scoring function for the trees. One such optimality criterion is *maximum likelihood* (ML) [12]. Finding the optimal tree under ML is known to be NP-hard [13]. Note that the number of possible unrooted tree topologies for *n* species, located at the tips, grows super-exponentially with *n*. Therefore, widely used tools for ML-based inference of phylogenies, such as RAxML [14] and PHYML [15], rely on heuristic search strategies for exploring the immense tree space.

The likelihood of each candidate tree topology *T* is calculated by computing the conditional likelihoods at each inner node of *T*. The conditional likelihoods are computed independently for each *site* (column in the MSA). They are computed via a post-order traversal of *T* starting from a virtual root. Note that, as long as the statistical model of evolution is time reversible (i.e. evolution occurred in the same way if followed forwards or backwards in time), the likelihood score is invariant with respect to where in *T* the virtual root has been placed.

For a node *k* with child nodes *i* and *j*, we compute the conditional likelihoods at *k* for each possible state (e.g. A, C, G, T for DNA data) as follows [12]:

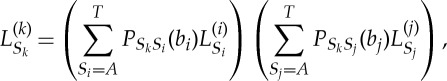

where 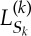 is the likelihood of observing the DNA nucleotide state *S*_*k*_ for the subtree rooted at *k*. The function *P*_*S*_*k*_*S*_*i*__(*b*_*i*_) gives the probability that base *S*_*k*_ evolved into base *S*_*i*_ after time *b*_*i*_ (the branch length from *i* to *k*). If *k* is a tip (leaf) and consists of a nucleotide, say *A*, then 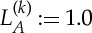 and 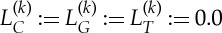. Finally, we compute the overall likelihood for a single site at the virtual root of the tree by multiplying the prior probabilities *π*_*x*_ (also called base frequencies) of observing a nucleotide state *x* with the likelihood of that state at the virtual root *r*

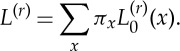



Once the likelihood for each site has been computed, the overall likelihood of the tree is the product over these per-site likelihoods. The tree topology is then modified using some tree alteration mechanisms (e.g. the RAxML search algorithm [14]), and the likelihood is computed again for the new tree. After a number of trees has been evaluated (usually millions), the tree with the best likelihood is returned.

In phylogenetic inference software, a common technique for optimizing the likelihood function, which typically consumes ≈95% of total execution time, is to eliminate duplicate *sites* (equivalent columns in the MSA). This is achieved by compressing identical sites into site patterns and assigning them a corresponding weight. This can be done because duplicate sites yield exactly the same likelihood iff they evolve under the same statistical model of evolution. When two sites are identical, this means that the leaves of the tree are labelled equally. Consider a forest of trees with the same topology, where, for each tree, the labels are defined by the molecular data stored at a particular site of the MSA and the position of the tips. Knowing equivalent subtrees within such a forest would allow someone to minimize the number of operations required to compute the likelihood of a phylogenetic tree. This can be seen as a generalization of the site compression technique.

*Our contribution*. In this article, we extend the series of results presented in [8,9] by introducing an algorithm that computes all subtree repeats in *unrooted unordered labelled* trees in linear time and space. The importance of our contribution is underlined by the fact that the presented algorithm can be easily modified to work on trees that do not satisfy some or any of the above assumptions on the tree structure, e.g. it can be applied to rooted, ordered or unlabelled trees. A preliminary version of this article appeared in [16].

## 2. Preliminaries

### (a) Basic definitions

An unrooted unordered tree is an undirected unordered acyclic-connected graph *T*=(*V*,*E*), where *V* is the set of nodes and *E* is the set of edges such that *E*⊂*V* ×*V* with |*E*|=|*V* |−1. The number of nodes of a tree *T* is denoted by |*T*|:=|*V* |. An *alphabet* *Σ* is a finite, non-empty set whose elements are called *symbols*. A *string* over an alphabet *Σ* is a finite, possibly empty, string of symbols of *Σ*. The length of a string *x* is denoted by |*x*|, and the concatenation of two strings *x* and *y* by *xy*. A tree *T* is *labelled* if every node of *T* is labelled by a symbol from some alphabet *Σ*. Different nodes may have the same label.

A *tree centre* of an unrooted tree *T*=(*V*,*E*) is the set of all vertices such that the greatest node distance to any leaf is minimal. An unrooted tree *T* has either one node that is a tree centre, in which case it is called a *central tree*, or two adjacent nodes that are tree centres, in which case it is called a *bicentral tree* [17]. Let 
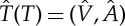
 be the rooted tree on 
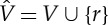
, where 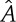 is defined such that 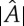 is minimal with 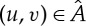 only if {*u*,*v*}∈*E* and each node other than *r* is reachable from one central point. If *T* is a bicentral tree, we add the additional root node *r* to *V* and add two edges to 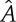, namely (*r*,*v*) and (*r*,*u*), where *v* and *u* are the central points of *T*—the edge between its two central points is not added. Otherwise, if *T* is a central tree, with tree centre *u*, we set *r*:=*u* and thus 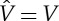.

We call *u*∈*V* a *child* of *v* iff 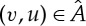. In this case, we call *v* the *parent* of *u* and define *parent*(*u*):=*v*. We call *u* and *u*^′^ *siblings* iff there exists a node 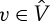 such that (*v*,*u*), 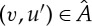. Note that under this definition two central points of a bicentral tree are siblings of each other.

The (rooted) subtree that contains node *v* as its root node, obtained by removing edge {*v*,*u*}, is denoted by 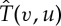. We consider only *full subtrees*, i.e. subtrees which contain all nodes and edges that can be reached from *v* when only the edge {*v*,*u*} is removed from the tree. The special case 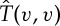 denotes the tree containing all nodes that is rooted in *v*. For simplicity, we refer to 
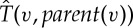
 as 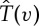. The *diameter* of an unrooted tree *T* is denoted by *d*(*T*) and is defined as the number of edges of the longest path between any two *leaves* (nodes with degree 1) of *T*. The *height of a rooted (sub)tree* 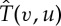 of some tree *T*, denoted by *h*(*v*,*u*), is defined as the number of edges on the longest path from the root *v* to some leaf of 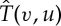. The *height of a node* *v*, denoted by *h*(*v*), is defined as the length of the longest path from *v* to some leaf in 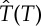.

For simplicity, in the rest of the text, we denote: a rooted unordered labelled tree by 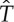, an unrooted unordered labelled tree by *T* and the rooted (directed) version of *T* by 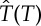, as defined above.

### (b) Subtree repeats

Two trees 
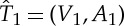
 and 
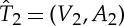
 are *equal*, denoted by 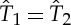, if there exists a bijective mapping *f*:*V*_1_→*V*_2_ such that the following two properties hold:

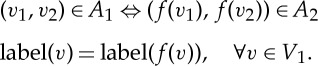

**Figure 1.**
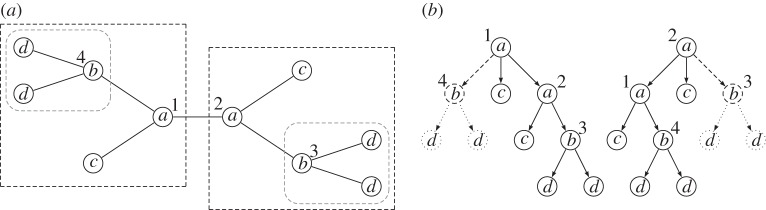
(*a*) An *unrooted tree* *T* consisting of 10 nodes; a *non-overlapping subtree repeat* *R*={(3,2),(4,1)} is marked with dashed rounded rectangles; another *non-overlapping subtree repeat* containing the trees 
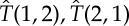
 is marked with dashed rectangles. (*b*) An *overlapping subtree repeat* *R*={(2,3),(1,4)} of *T* resulting from the deletion of the dashed edge and its corresponding dotted subtree. This is an overlapping subtree repeat since nodes 1 and 2—and the node labelled by *c*—are in both subtrees. A *total repeat* *R*={(1,1),(2,2)} of *T* can be obtained by keeping all the edges and rooting *T* in node 1 (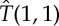) and node 2 (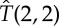), respectively.

A *subtree repeat* *R* in a tree *T* is a set of node tuples (*u*_1_,*v*_1_),…,(*u*_|*R*|_,*v*_|*R*|_), such that 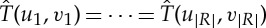. We call |*R*| the repetition *frequency* of *R*. If |*R*|=1 we say that the subtree 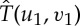 does not repeat. An *overlapping* subtree repeat is a subtree repeat *R*, where at least one node *v* is contained in all |*R*| trees. If no such *v* exists, we call it a *non-overlapping* subtree repeat. A *total repeat* *R* is a subtree repeat that contains all nodes in *T*, that is, *R*={(*u*_1_,*u*_1_),…,(*u*_|*R*|_,*u*_|*R*|_)}. See figure 1 in this regard.

In the following, we consider the problem of computing all such subtree repeats of an unrooted tree *T*.

## 3. Algorithm

The algorithm works in two stages: the forward/non-overlapping stage and the backward/overlapping stage. The forward stage finds all non-overlapping subtree repeats of some tree *T*. The backward stage uses the identifiers assigned during the forward stage to detect all overlapping subtree repeats, including total repeats.

### (a) The forward/non-overlapping stage

We initially present a brief description of the algorithmic steps. Thereafter, we provide a formal description of each step in algorithm 1. This algorithm resembles the one in [18] for deciding tree isomorphism.

In the following, we identify each node in the tree by a unique integer in the range of 1 to |*T*|. Such a unique integer labelling can be obtained, for instance, by a pre- or post-order tree traversal.

The basic idea of the algorithm can be explained by the following steps:

- Partition nodes by height.
- Assign a unique identifier to each label in *Σ*.
- For each height level starting from 0 (the leaves).
  1. For each node *v* of the current height level construct a string containing the identifier of the label of *v* and the identifiers of the subtrees that are attached to *v*.
  2. For each such string, sort the identifiers within the string.
  3. Lexicographically sort the strings (for the current height level).
  4. Find non-overlapping subtree repeats as identical adjacent strings in the lexicographically sorted sequence of strings.
  5. Assign unique identifiers to each set of repeating subtrees (equivalence class).

We will explain each step by referring to the corresponding lines in algorithm 1.

Partitioning the nodes according to their height requires time linear with respect to the size of the tree and is described in line 2 of algorithm 1. This is done using an array *H* of queues, where *H*[*i*], for all 0≤*i*≤⌊*d*(*T*)/2⌋, contains all nodes of height *i*. Thereafter, we assign a unique identifier to each label in *Σ* in lines 3–7. The main loop of the algorithm starts at line 8 and processes the nodes at each height level starting bottom-up from the leaves towards the central points. The main loop consists of four steps. First, a string is constructed for each node *v* which comprises the identifier for the label at *v* followed by the identifiers assigned to *u*_1_,*u*_2_,…,*u*_*c*_*v*__. The identifiers of *u*_1_,*u*_2_,…,*u*_*c*_*v*__ represent the subtrees 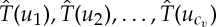, where *u*_1_,*u*_2_,…,*u*_*c*_*v*__ are the children of *v* (lines 11–16). Assume that this particular step constructs *k* strings *s*_1_,*s*_2_,…,*s*_*k*_.

In the next step, we sort the identifiers within each string. To obtain this sorting in linear time, we first need to remap individual identifiers contained as letters in those strings to the range [1,*m*]. Here, *m* is the number of unique identifiers in the strings constructed for this particular height, and the following property holds: 
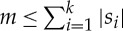
. We then apply a bucket sort to these remapped identifiers and reconstruct the ordered strings *r*_1_,*r*_2_,…,*r*_*k*_ (lines 17–20).

The next step for the current height level is to find the subtree repeats as identical strings. To achieve this, we lexicographically sort the ordered strings *r*_1_,*r*_2_,…,*r*_*k*_ (line 22), and check neighbouring strings for equivalence (lines 23–33). For each equivalence class 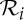 we choose a new, unique identifier that is assigned to the root nodes of all the subtrees in that class (lines 26 and 33). Finally, each set 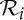 contains exactly the tuples of those nodes that are the roots of a particular non-overlapping subtree repeat of *T* and their respective parents.

Remapping from 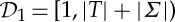 to 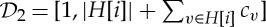 can be done using an array *A* of size |*T*|+|*Σ*|, a counter *m* and a queue *Q*. We read the numbers of the strings one by one. If a number *x* from domain 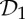 is read for the first time, we increase the counter *m* by one, set *A*[*x*]:=*m*, and place *m* in *Q*. Subsequently, we replace *x* by *m* in the string. In case a number *x* has already been read, that is, *A*[*x*]≠0, we replace *x* by *A*[*x*] in the string. When the remapping step is completed, only the altered positions in array *A* will be cleaned up, by traversing the elements of *Q*.

**Theorem 3.1 (Correctness)**

Given an unrooted tree T, algorithm 1 correctly computes all non-overlapping subtree repeats.

**Proof.**

First note that if any two subtrees 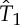 and 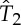 are repeats of each other, they must, by definition, be of the same height. So the algorithm is correct in only comparing trees of the same height. Additionally, non-overlapping subtree repeats of a tree *T* can only be of height ⌊*d*(*T*)/2⌋ or less, where *d*(*T*) is the diameter of *T*. Therefore, the algorithm is correct in stopping after processing all ⌊*d*(*T*)/2⌋+1 height classes, in order to extract all the non-overlapping subtree repeats. As the algorithm only extracts non-overlapping repeats, we define repeats to mean non-overlapping repeats for the rest of this proof. In addition, for simplicity, we consider the rooted version of *T* for the rest of this proof.

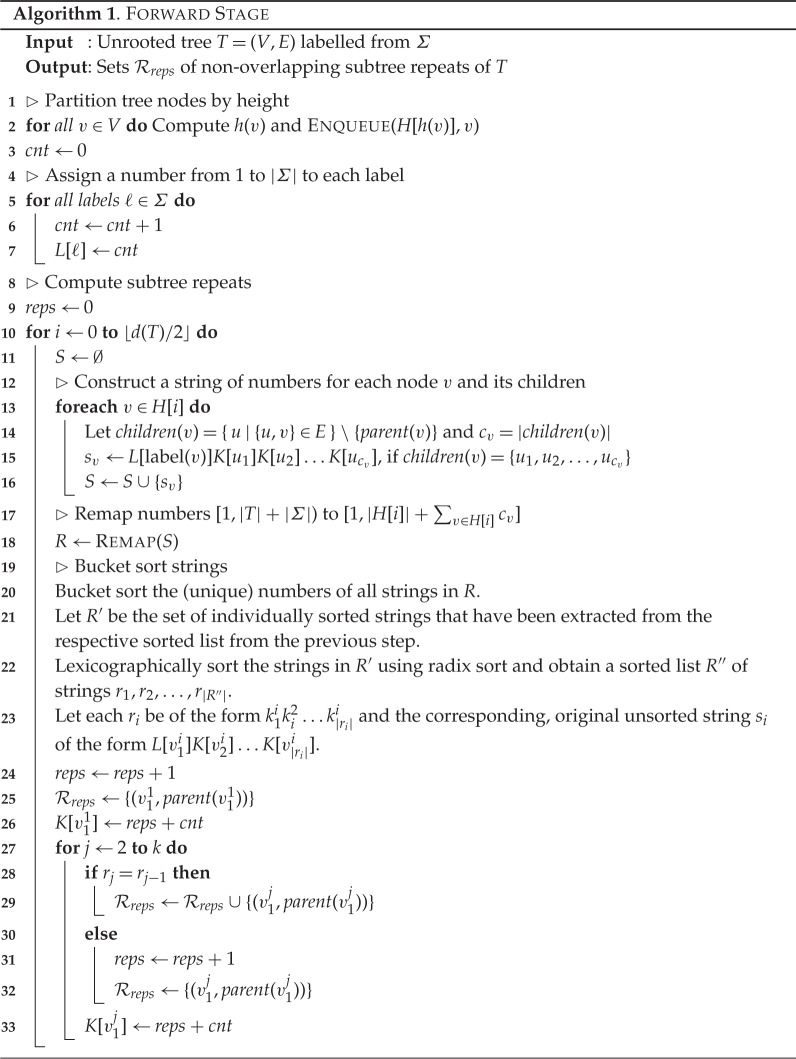

We show that the algorithm correctly computes all repeats for a tree of any height by induction. For the base case, we consider an arbitrary tree of height 1 (trees with height 0 are trivial). Any tree of height 1 only has the root node and any number of leaves attached to it. At the root we can never find a subtree repeat, so we need only to consider the next lower (height) level, that is, the leaf nodes. Any two leaves with identical labels will, by construction of the algorithm, be assigned the same identifiers and thus be correctly recognized as repeats of each other.

Now, assume that all (sub)trees of height *m*−1 have correctly been assigned with identifiers by the algorithm *and* that they are identical for two (sub)trees iff they are unordered repeats of each other.

Consider an arbitrary tree of height *m*+1. The number of repeats for the tree spanned from the root (node *r*) is always one (the whole tree). Now consider the subtrees of height *m*. The root of any subtree of height *m* must be a child of *r*. For any child of *r* that induces a tree of height smaller than *m*, all repeats have already been correctly calculated according to our assumption.

Two (sub)trees are repeats of each other iff the two roots have the same label and there is a one-to-one mapping from subtrees induced by children of the root of one tree to topologically equivalent subtrees induced by children of the root of the second tree. By the induction hypothesis, all such topologically equivalent subtrees of height *m*−1 or smaller have already been assigned identifiers that are unique for each equivalence class. Thus, deciding whether two subtrees are repeats of each other can be done by comparing the root labels and the corresponding identifiers of their children, which is exactly the process described in the algorithm. The approach used in the algorithm correctly identifies identically labelled strings since the order of identifiers has been sorted for a given height class. Thus, the algorithm finds all repeats of height *m* (and *m*+1 at the root).

**Theorem 3.2 (Complexity)**

*Algorithm 1 runs in time and space* 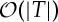.

**Proof.**

We prove the linearity of the algorithm by analysing each of the steps in the outline of the algorithm. Steps (i) and (ii) are trivial and can be computed in |*T*| and |*Σ*| steps, respectively. Note that |*Σ*|≤|*T*|.

The main for loop visits each node of *T* once. For each node *v* a string *s*_*v*_ is constructed which contains the identifier of the label of *v* and the identifiers assigned to the child nodes of *v*. Thus, each node is visited at most twice: once as parent and once as child. This leads to 2*n*−1 node traversals, where *n* is the number of nodes of *T*, since the root node is the only node that is visited exactly once. The constructed strings for a height level *i* are composed of the nodes in *H*[*i*] and their respective children. In total, we have 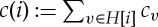 child nodes at a height level *i*, where *c*_*v*_ is the number of children of node *v*. Therefore, the total size of all constructed strings for a particular height level *i* is |*H*[*i*]|+*c*(*i*). Step (iii)(2) runs in linear time with respect to the number of nodes at each height level *i* *and* their children. This is because the remapping is computed in linear time with respect to |*H*[*i*]|+*c*(*i*). By the remapping, we ensure that the identifiers in each string are within the range of 1 to |*H*[*i*]|+*c*(*i*). Using bucket sort, we can then sort the remapped identifiers in time |*H*[*i*]|+*c*(*i*) for each height level *i*. Consequently, the identifiers in each string can be sorted in time |*H*[*i*]|+*c*(*i*) by traversing the sorted list of identifiers and positioning the respective identifier in the corresponding string on a first-read-first-place basis. This requires additional space |*H*[*i*]|+*c*(*i*) to keep track of which remapped identifier corresponds to which strings.

After remapping and sorting the strings, finding identical strings as repeats requires a lexicographical sorting of the strings. Strings that are identical form classes of repeats. Lexicographical sorting (using radix sort) requires time 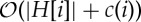 and at most space for storing |*T*|+|*Σ*| elements since the identifiers are in the range of 1 to |*T*|+|*Σ*|. This memory space needs to be allocated only once. Moreover, the elements that have been used are cleared/cleaned-up at each step via a queue as explained for the remapping function.

By summing over all height levels, we obtain 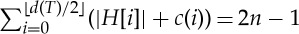. Thus, the total time over all height levels for each step described in the loop is 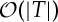. The overall time and space complexity of the algorithm are thus 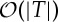.

**Figure 2.**
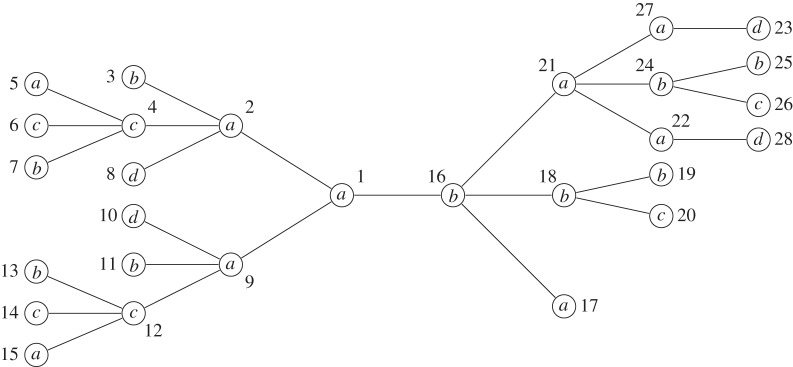
Graphical representation of tree *T*. The numbers denote the unique identifier assigned to each node by traversing *T*.

**Figure 3.**
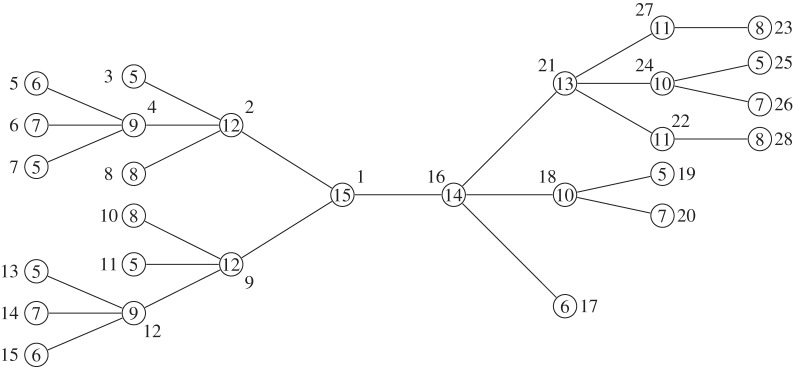
Graphical representation of tree *T* with the associated identifier for each node as assigned by algorithm 1.

We conclude this section with an example demonstrating algorithm 1. Consider the tree *T* from figure 2. The superscript indices denote the number associated with each node, which, in this particular example, correspond to a pre-order traversal of 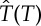 by designating node 1 as the root. Lines 1 and 2 partition the nodes of *T* in ⌊*d*(*T*)/2⌋+1 sets according to their height. The sets *H*[0]={3,5,6,7,8,10,11,13,14,15,17,19,20,23,25,26,28}, *H*[1]={4,12,18,22,24,27}, *H*[2]={2,9,21} and *H*[3]={1,16} are created. Lines 5–7 create a mapping between labels and numbers. *L*[*a*]=1, *L*[*b*]=2, *L*[*c*]=3 and *L*[*d*]=4. Table 1 shows the state of lists *S*,*R*,*R*′,*R*′′ during the computation of the main loop of algorithm 1 for each height level, where *S* is the list of string identifiers, *R* is the list of remapped identifiers, *R*′ is the list of individually sorted remapped identifiers and *R*′′ is the list *R*′ lexicographically sorted. Figure 3 depicts tree *T* with the respective identifiers for each node as assigned by algorithm 1.

**Table 1.** State of lists *S*,*R*,*R*′,*R*′′ for each height level and resulting sets 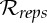 of non-overlapping subtree repeats.

height	step	process	repeats
0	strings: *S*	2,1,3,2,4,4,2,2,3,1,1,2,3,4,2,3,4	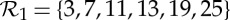
	remapping: *R*	1,2,3,1,4,4,1,1,3,2,2,1,3,4,1,3,4	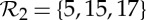
	sorting: *R*′	1,2,3,1,4,4,1,1,3,2,2,1,3,4,1,3,4	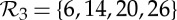
	repeats: *R*′′	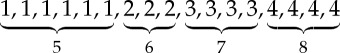	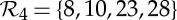
1	strings: *S*	3 6 7 5,3 5 7 6,2 5 7,1 8,2 5 7,1 8	
	remapping: *R*	1 2 3 4,1 4 3 2,5 4 3,6 7,5 4 3,6 7	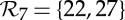
	sorting: *R*′	1 2 3 4,1 2 3 4,3 4 5,6 7,3 4 5,6 7	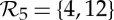
	repeats: *R*′′	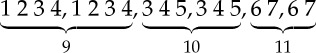	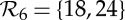
2	strings: *S*	1 5 9 8,1 8 5 9,1 11 10 11	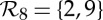
	remapping: *R*	1 2 3 4,1 4 2 3,1 5 6 5	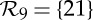
	sorting: *R*′	1 2 3 4,1 2 3 4,1 5 5 6	
	repeats: *R*′′	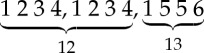	
3	strings: *S*	2 6 10 13,1 12 12	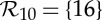
	remapping: *R*	1 2 3 4,5 6 6	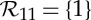
	sorting: *R*′	1 2 3 4,5 6 6	
	repeats: *R*′′	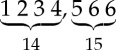	

### (b) The backward/overlapping stage

**Definition 3.3 (Sibling repeat)**

Given an unrooted tree *T*, two equal subtrees of 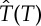 whose roots have the same parent are called a *sibling repeat*.

**Definition 3.4 (Child repeat—recursively defined)**

Given an unrooted tree *T*, two subtrees of 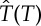 whose roots have the same identifiers and whose root’s respective parents are roots of trees in the same sibling or child repeat are called a *child repeat*.

Note that with these definitions we get that two trees with roots *u* and *v*, respectively, are child or sibling repeats of each other iff the unique path between nodes *u* and *v* is symmetrical with respect to the node identifiers of the nodes traversed on the path.

The two following lemmas illustrate why it is necessary and sufficient to know the identifiers from the forward stage to compute all overlapping subtree repeats.

**Lemma 3.5 (Sufficient conditions)**

Let *r* be the parent of *u* and *v*, where *u* and *v* are roots of a sibling repeat. Then the trees 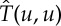 and 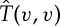 are elements of the same total repeat. The trees 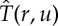 and 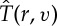 are elements of the same overlapping subtree repeat.

Let *u* and *v* be roots of a child repeat. Furthermore, let *r*_*u*_ and *r*_*v*_ be the parents of *u* and *v*, respectively. Then the trees 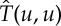 and 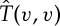 are elements of the same total repeat, and the trees 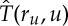 and 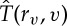 are elements of the same overlapping subtree repeat.

**Proof.**

Trivial, by inspection; see figure 2.

In figure 2, the trees 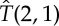 and 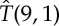 form a sibling repeat, thus the trees 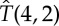 and 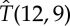 form a child repeat. From the sibling repeat, we get that 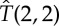 and 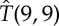 are elements of a total repeat, while 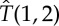 and 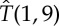 are within the same overlapping repeat. Analogously, for the child repeat we get the trees 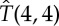 and 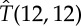 as total repeats and {(2,4),(9,12)} as an overlapping repeat.

Note that lemma 3.5 implies that all nodes of a subtree that is an element of an overlapping subtree repeat with repetition frequency |*R*| are roots of trees in overlapping repeat classes of frequency at least |*R*|.

**Lemma 3.6 (Necessary conditions)**

Any two trees that are elements of a total repeat must have been assigned the same identifiers at their respective roots during the forward stage, and be rooted in roots of either sibling or child repeats.

Any two trees that are elements of an overlapping subtree repeat, but not of a total repeat, must have been assigned the same identifiers at their respective roots during the forward stage, and be rooted in parents of roots of either sibling or child repeats.

**Proof.**

**Figure 4.**
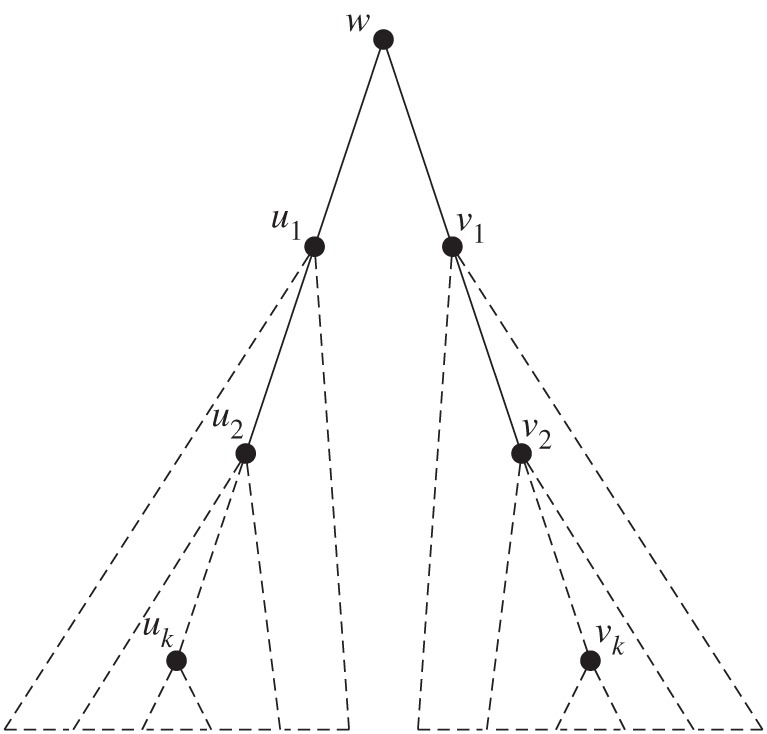
*T*(*v*_2_,*v*_*k*_)=*T*(*u*_2_,*u*_*k*_) is an overlapping repeat iff *T*(*u*_*k*_,*u*_2_)=*T*(*v*_*k*_,*v*_2_) is a child repeat, which is true iff *identifier*(*u*_*k*_)=*identifier*(*v*_*k*_), *identifier*(*u*_2_)= *identifier*(*v*_2_), *identifier*(*u*_1_)=*identifier*(*v*_1_).

We first look at the case of total repeats. Let 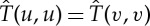. We now consider the unique path *p* between *u* and *v*. Obviously, for equality among these two trees to hold, the path must be symmetrical, which by recursion implies that *u* and *v* are roots of either sibling or child repeats (figure 4).

The case of other overlapping subtree repeats works just the same. Let 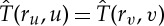 not be total, but an overlapping subtree repeat. These trees are obtained by removing a single edge from the tree: {*r*_*u*_,*u*} and {*r*_*v*_,*v*}, respectively. Let *p* be the path between *u* and *v*. Since the trees are elements of an overlapping subtree repeat, *r*_*u*_ and *r*_*v*_ must lie on this path. Additionally, since *r*_*u*_ and *r*_*v*_ are on the path from *u* to *v*, *h*(*v*)=*h*(*u*), and since any tree is acyclic, then *r*_*u*_ and *r*_*v*_ must be closer to the central points than *u* and *v*, respectively. Since there is an edge connecting *r*_*u*_ with *u* and *r*_*v*_ with *v* this means that *r*_*u*_ and *r*_*v*_ are parents of *u* and *v*, respectively. Again, the path *p* is symmetrical with respect to the node labels of nodes along the path, so *u* and *v* are roots of either sibling or child repeats.

Given these two lemmas, we can compute all overlapping subtree repeats by checking for sibling and child repeats. This can be done by comparing the identifiers assigned to nodes in the forward stage. The actual procedure of computing all overlapping subtree repeats is described in algorithm 2. Algorithm 2 takes as input an unrooted tree *T* that has been processed by algorithm 1, i.e. each node of tree *T* has already been assigned an identifier according to its non-overlapping repeat class.

First, the algorithm considers the rooted version of *T*, that is, 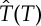. This is done since many operations and definitions rely on 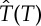. Next, we define a queue *Q*, whose elements are sets of nodes. Initially, *Q* contains only the set containing the root node of 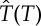 (line 2). Processing *Q* is done by dequeuing a single set of nodes at a time (lines 5–16). For a given set *U* of *Q*, the algorithm creates a set *I* containing the identifiers of children of all the nodes in *U*. Then, the algorithm remaps these identifiers to the range of [1,|*I*|] constructing a new set *I*′ (line 12). Then, we construct a list *C* of tuples, such that each tuple contains the remapped identifier of a child and the corresponding node. Therefore, we can use bucket sort to sort these tuples by the remapped identifiers in time linear in the cardinality of *I*.

We are now in a position to apply lemmas 3.5 and 3.6. By lemma 3.6, finding sibling and child repeats is done by creating sets of nodes with equivalent identifiers in *C* (line 18). This can be easily done because of the sorting part of the algorithm. These sets are then enqueued in *Q*, and, by lemmas 3.5 and 3.6, all resulting subtree repeats (overlapping and total) are, thus, created (lines 21–22). Hence we immediately obtain the following result.


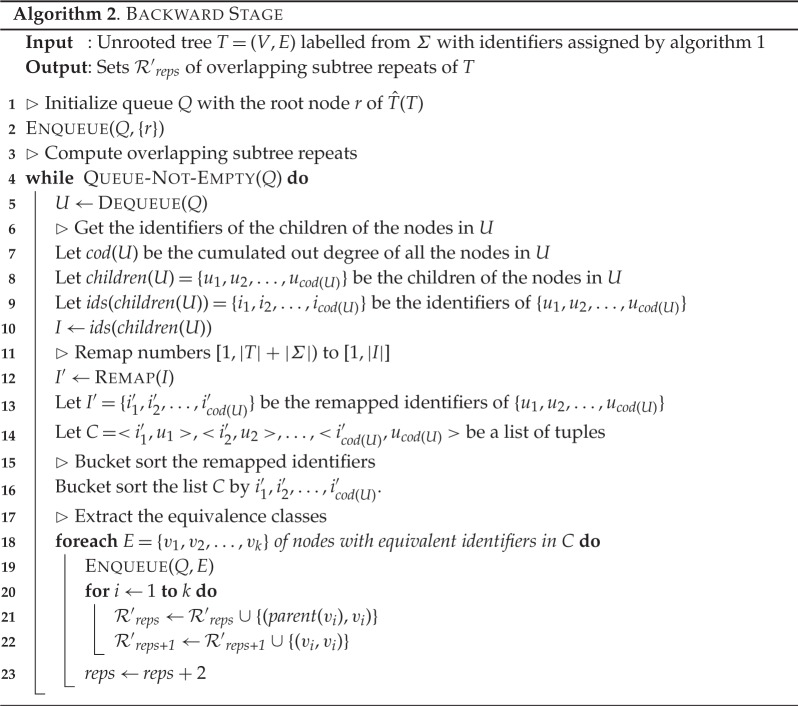

**Theorem 3.7 (Correctness)**

Given an unrooted tree T with identifiers assigned by algorithm 1, algorithm 2 correctly computes all overlapping subtree repeats, including total repeats.

Algorithm 2 enqueues each node of *T* once. For each enqueued node, a constant number of operations is performed. Therefore, we get the following result.

**Theorem 3.8 (Complexity)**

*Algorithm 2 runs in time and space* 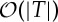.

## 4. Properties

In this section, we provide properties which could potentially be used to speed up an implementation of the above algorithms.

**Property 4.1 (Trivial path)**

If we find a non-overlapping subtree with repetition frequency 1, no node that lies on the path from the root of that subtree to the furthest central point (including the central point itself ) can have a repetition frequency other than 1 for non-overlapping subtree repeats rooted in this node. We call this path the trivial path.

**Proof.**

The proof is trivial. Assume that some node *v* on the trivial path would induce a non-overlapping subtree repeat with frequency higher than 1. By definition, all subtrees of the subtree rooted in *v* must be contained in all subtrees in this repeat. In particular, the original subtree with repetition frequency 1.

The implications for implementations are obvious. Any time we encounter a subtree with repetition frequency 1, we can mark all nodes on the trivial path as *trivial*, and add them to their own repeat class.

**Property 4.2 (Inclusion of trivial path)**

All trees from overlapping subtree repeats with repetition frequency higher than 1 must contain all nodes that lie on any trivial path.

**Proof.**

The proof is trivial. We prove the property by contradiction. Let *v* be a node on the trivial path. Then, by construction of overlapping subtree repeats only a single subtree in the repeat contains *v*. However, since *v* is on the trivial path, there must be a subtree without repeats induced by it. That is, no other subtree in the same overlapping repeat can have this subtree included, which contradicts the equality among trees.

## 5. Conclusion

We presented a simple and time-optimal algorithm for computing all full subtree repeats in unrooted unordered labelled trees, and showed that the running time of our method is linear with respect to the size of the input tree.

The presented algorithm can easily be modified to operate on trees that do not satisfy some or any of the aforementioned assumptions on the tree structure.

— *Rooted trees*: in a rooted tree 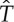, only non-overlapping repeats can occur. Therefore, it is sufficient to apply algorithm 1 with the following modifications: first, we define 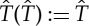; second, the main for loop must iterate over the height of 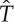, instead of depending on its diameter.
— *Ordered trees*: if for a node the order of its adjacent nodes is relevant, i.e. the tree is ordered, the bucket sort procedures in algorithms 1 and 2 must be omitted. Additionally, sibling repeats must not be merged in line 19 of algorithm 2 but rather be enqueued separately.
— *Unlabelled trees*: trivially, an unlabelled tree can be seen as a labelled tree with a single uniform symbol assigned to all nodes.

Algorithm 1 can also be used to compute subtree repeats over a *forest* of rooted unordered trees. The method is the same as for the case of a single tree. The method reports all subtree repeats by clustering the identifiers of equal subtrees from all trees in the forest into an equivalence class. The correctness of this approach can be trivially obtained by connecting the roots of all trees in the forest with a virtual root node, and applying the algorithm to this single tree. This solves the problem involved in the concrete application scenario discussed in §1.

Algorithm 1 can also be directly applied to solve the *maximal leaf-agreement isomorphic descendant subtree* (MLAIDS) problem [19]. MLAIDS is defined as follows: given a set of *k* phylogenetic (evolutionary) trees, find *k* maximal subtrees from the given trees, such that the leaves as well as the structure of the subtrees are equal.

## Footnotes

1 http://www.1kite.org/http://www.1kite.org/.
